# A MEIG1/PACRG complex in the manchette is essential for building the sperm flagella

**DOI:** 10.1242/dev.119834

**Published:** 2015-03-01

**Authors:** Wei Li, Waixing Tang, Maria E. Teves, Zhengang Zhang, Ling Zhang, Hongfei Li, Kellie J. Archer, Darrell L. Peterson, David C. Williams, Jerome F. Strauss, Zhibing Zhang

**Affiliations:** 1 Department of Obstetrics and Gynecology, Virginia Commonwealth University, Richmond, VA 23298, USA; 2 Department of Otorhinolaryngology, University of Pennsylvania, Philadelphia, PA 19104, USA; 3 Department of Infectious Diseases, Tongji Medical College, Huazhong University of Science and Technology, Wuhan, Hubei 430030, China; 4 School of Public Health, Wuhan University of Science and Technology, Wuhan, Hubei 430081, China; 5 Department of Biostatistics, Virginia Commonwealth University, Richmond, VA 23298, USA; 6 Department of Biochemistry & Molecular Biology, Virginia Commonwealth University, Richmond, VA 23298, USA; 7 Department of Pathology and Laboratory Medicine, University of North Carolina, Chapel Hill, NC 27599, USA

**Keywords:** Cargo transport, Manchette, Sperm flagella, Spermiogenesis

## Abstract

A key event in the process of spermiogenesis is the formation of the flagella, which enables sperm to reach eggs for fertilization. Yeast two-hybrid studies revealed that meiosis-expressed gene 1 (MEIG1) and Parkin co-regulated gene (PACRG) interact, and that sperm-associated antigen 16, which encodes an axoneme central apparatus protein, is also a binding partner of MEIG1. In spermatocytes of wild-type mice, MEIG1 is expressed in the whole germ cell bodies, but the protein migrates to the manchette, a unique structure at the base of elongating spermatid that directs formation of the flagella. In the elongating spermatids of wild-type mice, PACRG colocalizes with α-tubulin, a marker for the manchette, whereas this localization was not changed in the few remaining elongating spermatids of *Meig1*-deficient mice. In addition, MEIG1 no longer localizes to the manchette in the remaining elongating spermatids of *Pacrg*-deficient mice, indicating that PACRG recruits MEIG1 to the manchette. PACRG is not stable in mammalian cells, but can be stabilized by MEIG1 or by inhibition of proteasome function. SPAG16L is present in the spermatocyte cytoplasm of wild-type mice, and in the manchette of elongating spermatids, but in the *Meig1* or *Pacrg*-deficient mice, SPAG16L no longer localizes to the manchette. By contrast, MEIG1 and PACRG are still present in the manchette of *Spag16L*-deficient mice, indicating that SPAG16L is a downstream partner of these two proteins. Together, our studies demonstrate that MEIG1/PACRG forms a complex in the manchette and that this complex is necessary to transport cargos, such as SPAG16L, to build the sperm flagella.

## Abstract (precis)

**Summary:** In the manchette, a structure at the base of the elongating spermatid, the proteins MEIG1 and PACRG act in a complex to control cargo transport and direct formation of the flagellum.

## INTRODUCTION

Mouse meiosis-expressed gene 1 (*Meig1*) was originally identified in a search for mammalian genes involved in meiotic processes (Don and Wolgemuth, 1992). Earlier studies suggested a role for MEIG1 in meiosis, and two *Meig1* cDNAs encoding the same protein were identified. The 0.75 kb transcript was highly abundant in the testis and began to accumulate in testes at day 8-9 of postnatal development (P8-9), coincident with the entry of germ cells into meiosis. MEIG1 is most abundant at P14 and subsequent stages, when the spermatocytes have entered the pachytene stage (Don et al., 1994). Dimerization and phosphorylation/dephosphorylation reactions have been proposed to regulate the function of MEIG1 during meiosis (Chen-Moses et al., 1997; Ever et al., 1999). The MEIG1 protein appears to form dimers of 31 kDa and 32 kDa, and the 31 kDa dimeric form enters the nucleus during the first meiotic prophase and binds to the meiotic chromatin (Steiner et al., 1999). It has been also reported that *Meig1* mRNA is not only expressed in germ cells, but also in somatic cells (Ever et al., 1999; Steiner et al., 1999).

MEIG1 was subsequently identified as a binding partner of mouse sperm-associated antigen 16 (SPAG16) (Zhang et al., 2004). The mouse *Spag16* gene is the ortholog of the *Chlamydomonas PF20* gene, which encodes a single protein localized to the axonemal central apparatus, where it regulates flagellar motility (Smith and Lefebvre, 1997). However, the mouse *Spag16* gene encodes two major transcripts, which are expressed in different patterns during spermatogenesis, yielding proteins of 71 and 35 kDa, respectively (Zhang et al., 2004). Both proteins contain contiguous WD repeats in their C termini. The 71 kDa protein is incorporated into the central apparatus and regulates sperm motility (Zhang et al., 2006), whereas the 35 kDa protein is also present in the nucleus, and is believed to play a role in the regulation of spermatogenesis (Zhang et al., 2004). Interaction between MEIG1 and SPAG16 further suggests a role of MEIG1 in male germ cell function.

Potential functions of MEIG1 in spermatogenesis/ciliogenesis were also suggested by several other studies. In heat shock transcription factor 2 mutant mice, *Meig1* expression is strongly reduced, which may lead to impaired spermatogenesis and reduced fertility (Wang et al., 2003). Owing to its abundant expression in tissues rich in highly ciliated cells, such as testis, lung and olfactory sensory neurons, *Meig1* is predicted to be important for ciliary development and/or function (McClintock et al., 2008). The role of MEIG1 in germ cells was proven by two independent studies conducted in two different laboratories using different *Meig1* mutant mouse models. We discovered that there are at least seven *Meig1* transcripts, and all of the seven transcripts encode the same protein. When *Meig1* was disrupted globally by crossing the floxed *Meig1* mice to *CMV-*Cre transgenic mice, *Meig1*-deficient males were completely infertile. Unexpectedly, the mutant mice had no obvious defect in meiosis, but were sterile as a result of impaired spermatogenesis at the stage of elongation and condensation (Zhang et al., 2009). Another laboratory generated a conventional *Meig1* mutant model. Consistent with the conditional mouse model, the mutant males were infertile. Seminiferous tubules in *Meig1*-null males contained all early stages of spermatogenesis, up to elongating spermatids, but mature elongated spermatids were absent (Salzberg et al., 2010). Both laboratories discovered that MEIG1 regulates the final step of spermatogenesis, spermiogenesis. Even though *Meig1* is also present in somatic cells in the testis (Ever et al., 1999; Bouma et al., 2007), we discovered that MEIG1 regulates spermatogenesis through its role in germ cells, but not in Sertoli cells (Teves et al., 2013). It has been reported that *Meig1* is also expressed in embryonic mouse ovary (Don et al., 1994; Chen-Moses et al., 1997), and *MEIG1* mRNA expression is significantly altered in individuals with premature ovarian failure (Ledig et al., 2010). However, *Meig1*-deficient females did not show a significant reproductive phenotype (Salzberg et al., 2010).

To investigate the mechanisms of MEIG1 function, a yeast two hybrid screen was conducted, and the major binding partner identified was PACRG (Zhang et al., 2009), a gene for which is located on chromosome 17 and co-regulated with *Parkin* (West et al., 2003). *Pacrg* was found to be the gene associated with the *quaking^viable^* (*qk^v^*) mutation – a 1.17 Mb deletion on chromosome 17 that causes a male infertility phenotype mirroring that of *Meig1* mutants (Bennett et al., 1971; Lockhart et al., 2004; Lorenzetti et al., 2004). In *Chlamydomonas reinhardtii*, PACRG has been shown to be a component of the centriole/basal bodies (Keller et al., 2005), whereas in *Trypanosoma brucei*, the two PACRG homologs localize along the full length of the axoneme. Axoneme structure was disrupted when both proteins were ablated by RNA interference knockdown, resulting in slow growth and paralysis of the flagellum (Gadelha et al., 2006). Moreover, a recent study demonstrated that variation in the human *PACRG* promoter was a risk factor associated with azoospermia (Wilson et al., 2010). Like MEIG1 protein, the exact mechanism of PACRG action is currently unknown. Given the shared phenotype of male mice with mutations in the two genes, and the strong interaction between the two proteins, we hypothesize that MEIG1 and PACRG form a complex essential for spermiogenesis.

In the *Meig1*-deficient mice, transmission electron microscopy revealed abnormal nucleus condensation and impaired flagellogenesis associated with a disrupted manchette structure (Zhang et al., 2009). The manchette is a transient microtubular structure assembled concurrently with the elongation and condensation of the spermatid nucleus and growth of the centrosome-derived axoneme. The appearance and disappearance of the manchette are likely related to the dynamic morphological changes in the spermatids during spermiogenesis (Kierszenbaum and Tres, 2004; O'Donnell and O'Bryan, 2014). The timing of manchette development is very precise; it appears during early elongation and disappears when the elongation and condensation processes of the spermatid nucleus approach completion. The manchette is composed of post-translationally modified α-/β-tubulin isoforms, actin, keratins 5 and 9, and motor and non-motor proteins (Li et al., 2014; Nozawa et al., 2014; Qi et al., 2013; Hayasaka et al., 2008; Mochida et al., 2000; Kato et al., 2004; Moreno and Schatten, 2000; Navolanic and Sperry, 2000). It has been proposed that the manchette is a key structure in the regulation of nucleus condensation and tail formation in spermatids (Kierszenbaum et al., 2007; Kierszenbaum, 2002).

In this study, we characterized localization of MEIG1, PACRG and SPAG16L in male germ cells of wild-type and mutant mice, and discovered that all the three proteins are present in the manchette of condensing/elongating spermatids. MEIG1 is expressed in the whole cell body in spermatocytes, whereas subsequent migration to the manchette coincides with and is dependent upon expression of PACRG. The localization of SPAG16L in the manchette depends on MEIG1 and PACRG, whereas manchette localization of MEIG1 and PACRG does not depend on SPAG16L. Our studies demonstrate that MEIG1/PACRG form a complex in the manchette, and this complex is essential to transport sperm flagella proteins, such as SPAG16L, to build sperm flagella.

## RESULTS

### Analysis of testicular histology of *Meig1*-deficient mice during the first wave of spermatogenesis

**Fig. 1.**
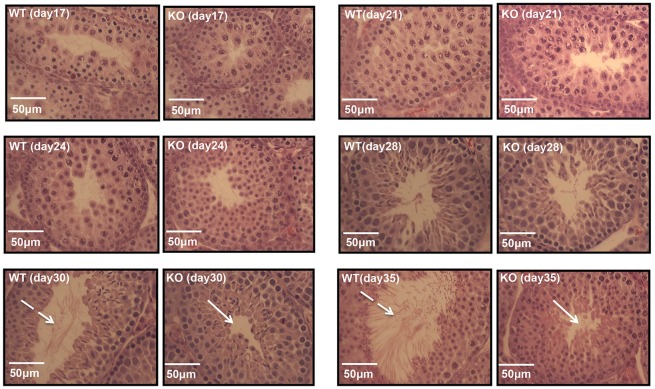
**Dynamic analysis of testicular histology of *Meig1*-deficient mice during the first wave of spermatogenesis.** Representative testicular sections stained with Hematoxylin and Eosin from wild-type (WT) and *Meig1*-deficient (KO) mice at the indicated ages. Testicular histology in *Meig1*-deficient mice is still normal 28 days after birth, but after this time point, when spermatids enter the condensation/elongation stage, they cease differentiation. The white dashed arrows indicate the released germ cells in the lumen of seminiferous tubules of wild-type mice at day 30 and 35 after birth, the white arrows indicate an empty lumen of the seminiferous tubules of *Meig1*-deficient mice at the same age.

In adult *Meig*1-deficient mice, spermatogenesis is arrested in spermiogenesis (Zhang et al., 2009). To further examine when the testes of *Meig1*-deficient mice undergo pathological changes during the first wave of spermatogenesis, testes from wild-type and *Meig1*-deficient mice were collected at different ages, from day 17 to day 35 after birth, and tissue sections were stained with H&E. Testicular histology in *Meig1*-deficient mice remains normal until 28 days after birth, but after this time point, when spermatids enter the condensation/elongation stage, they cease differentiation (Fig. 1).

### Gene expression profiles are comparable between wild-type and *Meig1*-deficient mice

To compare gene expression level between wild-type and *Meig1*-globally deficient mice in the testis, DNA microarray studies were conducted using total testicular RNA isolated from 22- and 28-day-old wild-type and *Meig1*-deficient mice, when morphology is comparable between the two groups of mice. At both time points, only a few genes were differentially expressed between wild-type and *Meig1*-deficient mice, even when using a generous *P*-value threshold of 0.01 (supplementary material Fig. S1 and Table S1). However, validation of some of these genes (e.g. *Arl5a*) by q-PCR and western blot analysis showed no differences between wild-type and *Meig1*-deficient mice.

### MEIG1 localization in mouse testis and mixed germ cells

**Fig. 2.**
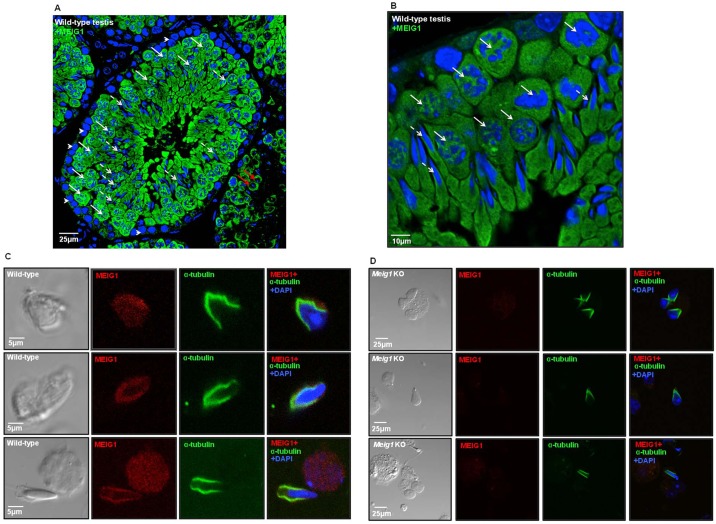
**MEIG1 localization in male germ cells.** (A,B) Testicular sections. (C,D) Isolated mixed germ cells. In the testicular section, the MEIG1 protein was labeled green. The arrowheads indicate spermatogonia, the white arrows indicate spermatocytes, the red arrows indicate round spermatids, and dashed arrows indicate the elongating spermatids. MEIG1 is not expressed in the spermatogonia; it is present in the whole cell body of spermatocytes and round spermatids, but migrates to the manchette of elongating spermatids. (C,D) α-Tubulin is still present around the nuclei in the remaining elongating spermatids of *Meig1*-deficient mice. MEIG1 is missing in all the germ cells, but localization of α-tubulin remains intact.

To understand the mechanism of action of MEIG1, we conducted immunofluorescence studies on testicular sections from adult wild-type mice to track localization of the protein. Using an affinity-purified MEIG1 antibody, we discovered that MEIG1 is not expressed in the spermatogonia. It is present in the whole cell body of spermatocytes, only around the nuclei of elongating spermatids (Fig. 2A,B). Localization of MEIG1 was further examined in mixed germ cells isolated from adult wild-type mice. The protein is visualized throughout the cell body in spermatocytes and early spermatids, but gradually concentrates in the manchette during the elongation/condensation process, and colocalizes with α-tubulin, a manchette marker (Fig. 2C). Mixed germ cells from *Meig1*-deficient mice were also double stained with an anti-MEIG1 antibody and an anti-α-tubulin antibody. MEIG1 is missing in all stages of germ cells, whereas α-tubulin is still present and labels the manchette structure (Fig. 2D).

### Mouse *Pacrg* gene expression is under post-transcriptional control and the translated protein is located in the manchette of elongating spermatids

**Fig. 3.**
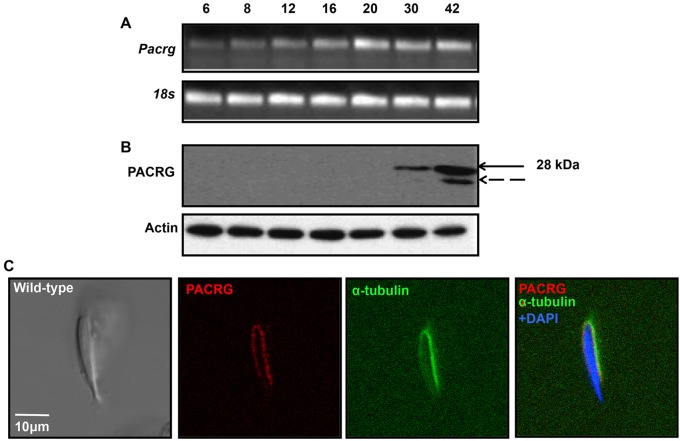
**Mouse *Pacrg* gene is under post-transcriptional control and the translated protein is on the manchette of elongating spermatids of wild-type mice.** Analysis of mouse *Pacrg* mRNA (A) and protein expression (B) during the first wave of spermatogenesis. mRNA is present as early as day 6 after birth, but the protein is not expressed until day 30 after birth, when germ cells enter the condensation/elongation stage. (C) PACRG protein is localized in the manchette of elongating spermatids. PACRG colocalizes with α-tubulin, a manchette marker. The arrow in B indicates the full-length PACRG protein, the dashed arrow pointing to a band only present in the day 42 testis may represent a degraded product from the full-length protein.

We have previously reported that PACRG is the major binding partner of the MEIG1 protein. To identify binding partners of PACRG, we performed a yeast two-hybrid analysis using mouse full-length PACRG as bait. Among 150 positive cDNAs sequenced, 100 encoded MEIG1. Thus, MEIG1 is also the major binding partner of the PACRG protein. To further investigate this gene, *Pacrg* mRNA and protein expression was examined in the testis of wild-type mice during the first wave of spermatogenesis. *Pacrg* mRNA is detectable by RT-PCR from day 6 after birth (Fig. 3A). However, the translated protein is not detectable by western blotting until day 30 after birth (Fig. 3B). To determine PACRG localization in mouse testis, immunofluorescence staining was conducted. Consistent with the western blot result, PACRG is expressed in the post-meiotic germ cells, and only in the region around nuclei of the elongating spermatids, where the manchette localizes (supplementary material Fig. S2A). To further verify its localization, mixed germ cells were isolated from adult mice, and these cells were double stained with anti-PACRG antibody and anti-α-tubulin antibody. Consistent with the results from testis sections, PACRG protein is present around the nuclei, and colocalizes with α-tubulin in the elongating spermatids, indicating that it is indeed localized in the manchette (Fig. 3C).

### PACRG protein is not stable, but can be stabilized by MEIG1 or inhibition of proteasome function

**Fig. 4.**
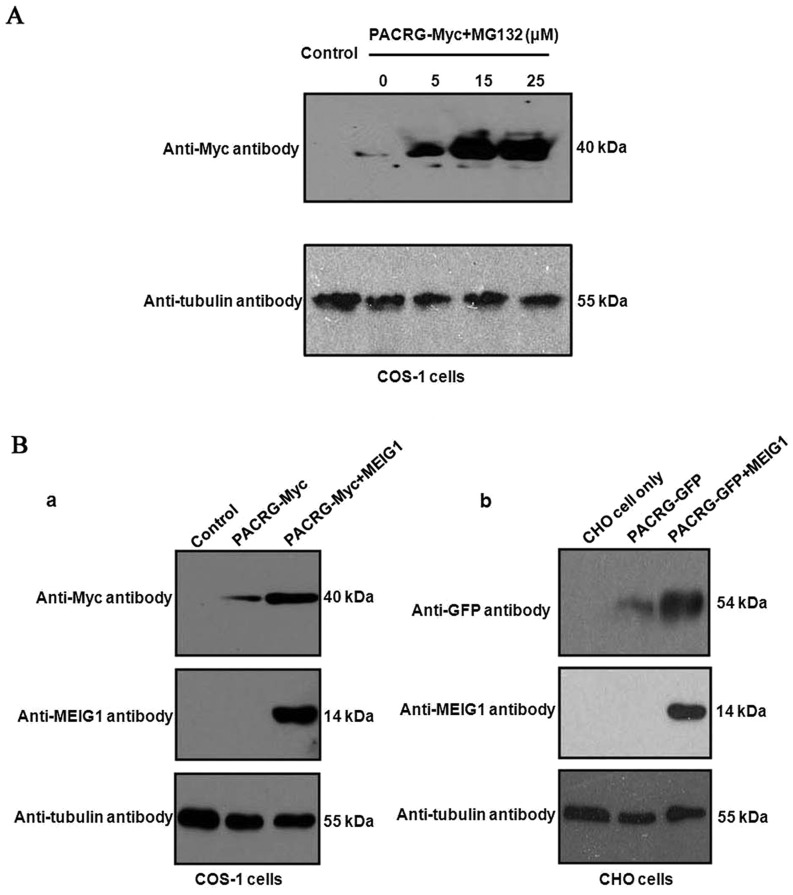
**PACRG protein is not stable but can be stabilized by inhibition of proteasome function or MEIG1.** (A) The proteasome inhibitor MG132 significantly increases mouse PACRG expression in transfected COS-1 cells. PACRG-Myc expression was increased by MG132 in a dose-dependent way. (B) MEIG1 increases PACRG expression. (a) Mouse PACRG-Myc expression is increased in the presence of MEIG1 in transfected COS-1 cells in transient expression experiment. (b) CHO cells. Stable expression of PACRG-GFP in cells that were transfected with a MEIG1 expression plasmid. PACRG expression was increased in the presence of MEIG1.

It has been reported that human PACRG abundance is regulated, in part, by the ubiquitin-proteasome system (Taylor et al., 2012, 2007). Mouse *Pacrg* mammalian expression plasmids were constructed, and PACRG protein expression was examined in mammalian cells. In transfected COS-1 cells, PACRG expression was significantly increased by the proteasome inhibitor, MG132 (Fig. 4A).

MEIG1 is a strong binding partner of PACRG. To test whether MEIG1 stabilizes PACRG, mouse PACRG was expressed in COS-1 cells with or without co-expression of MEIG1, and the PACRG expression levels were evaluated by western blotting. Co-expression of MEIG1 led to increased levels of PACRG protein only when equivalent amounts of *Pacrg* expression plasmid were transfected (Fig. 4Ba). To further validate this observation, a PACRG stable cell line was generated by transfection with a PACRG/pEGFP-C1 plasmid. When the cells were transfected with a *Meig1* expression plasmid, the expression level of GFP-tagged PACRG was significantly increased (Fig. 4Bb). 

### MEIG1 localization in the manchette is dependent upon PACRG

**Fig. 5.**
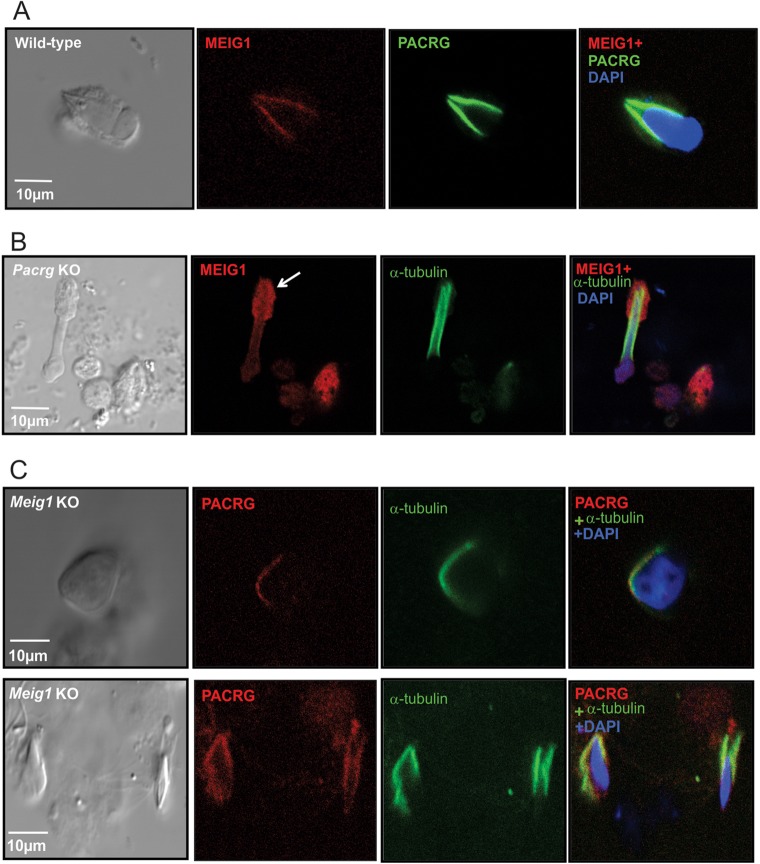
**MEIG1 localization on the manchette is dependent on PACRG protein.** (A) MEIG1 and PACRG colocalize on the manchette of elongating spermatids. (B) MEIG1 is present in the whole cell body of the remaining elongating spermatids of *Pacrg*-deficient mice. The white arrow indicates a surviving spermatid. (C) PACRG is still present in the manchette of the remaining elongating spermatids of *Meig1*-deficient mice.

Because both MEIG1 and PACRG localize in the manchette, and the two proteins interact, their colocalization was examined. A monoclonal antibody against PACRG was generated and mixed germ cells from adult wild-type mice were double stained with an anti-MEIG1 polyclonal antibody and the anti-PACRG monoclonal antibody. As expected, MEIG1 and PACRG colocalize in the manchette of elongating spermatids (Fig. 5A).

MEIG1 localization in male germ cells was examined in the *Pacrg*-deficient mice. As in the wild-type mice, MEIG1 was present in the whole cell body in spermatocytes, but in the remaining elongating spermatids, MEIG1 no longer localized only in the manchette, instead it was present in the whole cell body (Fig. 5B).

PACRG localization was examined in the *Meig1*-deficient mice. As in the wild-type mice, PACRG still localizes in the manchette of remaining elongating spermatids of *Meig1*-deficient mice (Fig. 5C). The same result was obtained when PACRG localization was analyzed in the testicular sections from the *Meig1*-deficient mice (supplementary material Fig. S2B), indicating that PACRG localization is not dependent on MEIG1.

### The manchette localization of SPAG16L, a sperm flagella central apparatus protein, in elongating spermatids is dependent upon MEIG1 and PACRG

**Fig. 6.**
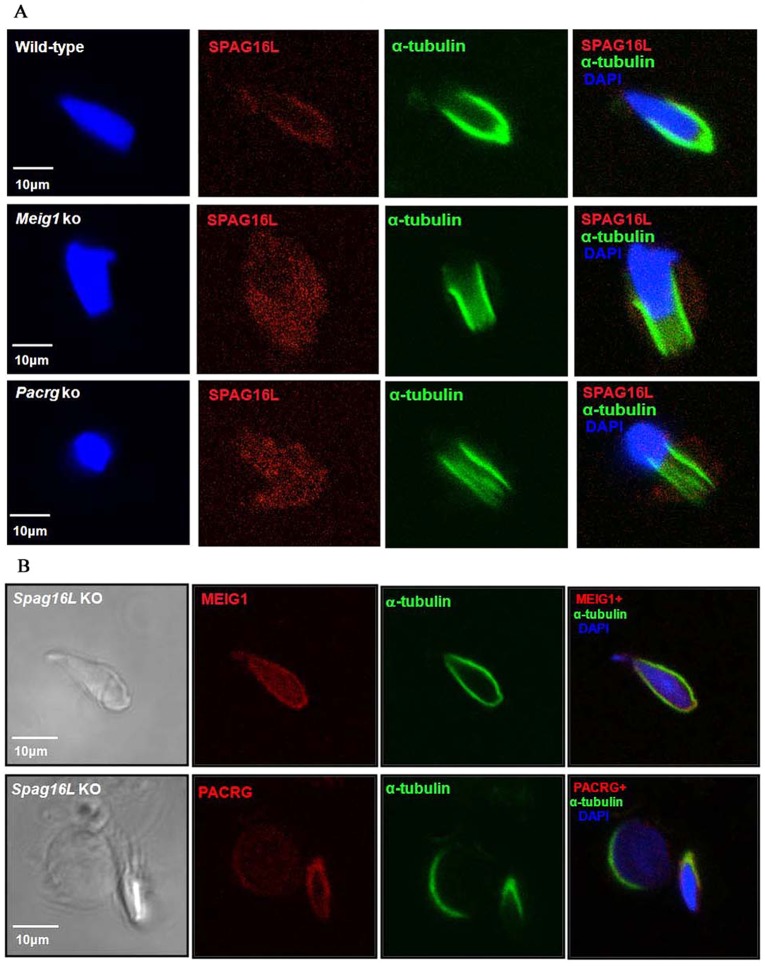
**Manchette localization of SPAG16L, a sperm flagella central apparatus protein, in the elongating spermatids is dependent on MEIG1 and PACRG.** (A) SPAG16L localization in the manchette is dependent on MEIG1 and PACRG. SPAG16L is present only in the manchette of elongating spermatids of wild-type mice (upper panel), but in the remaining elongating spermatids of *Meig1* and *Pacrg*-deficient mice, its localization is diffused in the whole cells. (B) MEIG1 (upper panel) and PACRG (lower panel) localization in the elongating spermatids of *Spag16L*-deficient mice is not different from wild-type mice.

MEIG1 interacts with the C-terminal region of SPAG16 protein (Zhang et al., 2004). The meiotically expressed SPAG16L is localized in the cytoplasm of spermatocytes (Zhang et al., 2004; Nagarkatti-Gude et al., 2011) and subsequently incorporated into the sperm flagella central apparatus, and is essential for sperm motility. To determine whether SPAG16L redistribution is dependent on MEIG1, we examined localization of SPAG16L in the elongating spermatids isolated from wild-type mice and *Meig1*-deficient mice. Not surprisingly, even though SPAG16L is localized in the cytoplasm of spermatocytes, in the elongating spermatids, it is localized in the manchette (upper panel of Fig. 6A). However, SPAG16L is no longer concentrated in the manchette of elongating spermatids from *Meig1*-deficient mice, but instead it is present in the whole cytoplasm (middle panel of Fig. 6A; supplementary material Fig. S3A,B). SPAG16L localization was also examined in the *Pacrg*-deficient mice. Similarly, SPAG16L is present throughout the whole cell body of the surviving elongating spermatids of *Pacrg*-deficient mice (lower panel of Fig. 6A; supplementary material Fig. S3C). However, the localization of both MEIG1 and PACRG in the elongating spermatids is not changed in the *Spag16L*-deficient mice (Fig. 6B).

Even though SPAG16L localization was dramatically changed in the elongating spermatids of *Meig1* and *Pacrg*-deficient mice, its localization in early stages germ cells was not altered in the two mutant mice (supplementary material Fig. S4).

## DISCUSSION

We previous analyzed adult *Meig1*-deficient mice, and discovered that spermatogenesis is arrested in spermiogenesis, when germ cells enter the condensation/elongation stage (Zhang et al., 2009). By analyzing the testis histology throughout the first wave of spermatogenesis, it was found that germ cells stop differentiation at day 28 after birth. This observation is consistent with our findings in testes from adult *Meig1*-deficient mice.

Dramatic testicular morphological changes in the *Meig1*-deficient mice may be due to altered gene expression. Chen-Moses reported that MEIG1 is expressed in the nuclei of spermatocytes and binds DNA (Chen-Moses et al., 1997). We hypothesized that MEIG1 might regulate expression of a suite of genes that are essential for germ cell development. We have previously analyzed mRNA and protein expression of representative genes whose translated proteins are present in the nuclei and flagella, and found no difference in their expression levels. To compare gene expression level between wild-type and *Meig1*-globally deficient mice in the testis, DNA microarray studies were conducted. Results from DNA array studies coupled with q-PCR and western blot analyses do not support the conclusion that MEIG1 has a role in the regulation of gene expression in the testis. The few differences in gene expression between wild-type and *Meig1*-deficient mice detected by DNA microarray studies may reflect an artifact arising from low baseline levels of expression.

Mouse *Meig1* gene has at least seven transcripts, some of which are expressed in multiple tissues, whereas others are only present in the testis (Zhang et al., 2009). In the testis, *Meig1* is expressed in somatic Sertoli cells and germ cells. Cell type-specific deletion of *Meig1* gene in the testis demonstrated that the primary role of MEIG1 in the regulation of spermatogenesis is through germ cells (Teves et al., 2013). Immunofluorescence studies on testicular sections and isolated mixed germ cells revealed that MEIG1 is present in the whole cell body in spermatocytes, but migrates to the manchette during the elongation/condensation process. Interestingly, in the *Meig1-*deficient mice, the major component, α-tubulin is still present and labels the manchette structure. This observation indicates that even though the manchette ultrastructure is disrupted in the germ cells of *Meig1*-deficient mice (Zhang et al., 2009), the backbone seems to be intact.

How is MEIG1 protein concentrated in the manchette in the elongating spermatids? We hypothesized that the localization is dependent upon another protein that is also present in the manchette. Given the strong interaction between MEIG1 and PACRG, and the fact that *Pacrg*-deficient male mice are infertile and the reproductive phenotype mirrors that of the *Meig1* mutant mice, we suggest that PACRG might be the protein that recruits MEIG1. Thus, mouse *Pacrg* was further characterized. *Pacrg* mRNA is expressed from day 6 after birth. However, the protein is not translated until day 30 after birth, much later than the mRNA, suggesting that the gene is under post-transcriptional control, either at the level of translation or perhaps post-translational stabilization, as discussed below. Like human PACRG, which is regulated, in part, by the ubiquitin-proteasome system (Taylor et al., 2012, 2007), mouse PACRG expression was significantly increased by the proteasome inhibitor MG132 in the transfected COS-1 cells, indicating that mouse PACRG is also regulated by the ubiquitin-proteasome system. Interestingly, in the transfected mammalian cells, co-expression of MEIG1 led to increased levels of PACRG protein, indicating that MEIG1 could stabilize PACRG protein levels. This is further evidence showing functional interaction between the two proteins. In mouse testis, PACRG is localized in the manchette and colocalizes with MEIG1 in the elongating spermatids. However, MEIG1 no longer localized in the manchette of the remaining elongating spermatids of *Pacrg*-deficient mice; on the contrary, PACRG is still present in the manchette of the remaining elongating spermatids of *Meig1*-deficient mice. Thus, PACRG is upstream of MEIG1 in localization to the manchette.

The MEIG1/PACRG complex seems to be essential for some proteins, such as SPAG16L to be assembled into sperm flagella and complete the spermatogenesis process. Like MEIG1, SPAG16L is also localized in the cytoplasm of spermatocytes; it is redistributed to the manchette of the elongating spermatids. However, SPAG16L is no longer concentrated in the manchette in either *Meig1* or *Pacrg*-deficient mice, and the fact that both MEIG1 and PACRG are still present in the manchette in the *Spag16L*-deficient mice, indicates that SPAG16L is a downstream protein of MEIG1/PACRG complex.

**Fig. 7.**
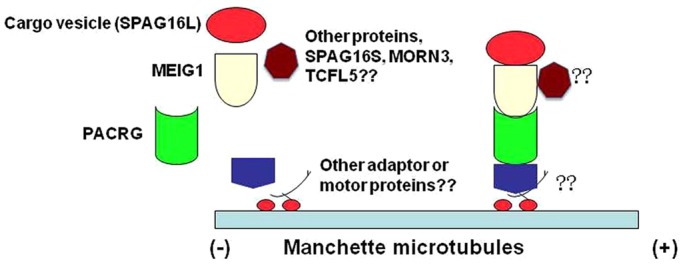
**Working model of the MEIG1/PACRG complex.** MEIG1 and PACRG form a complex, with PACRG being an upstream protein that recruits downstream MEIG1 to the manchette. PACRG associates with the manchette microtubules through other adaptors and molecular motor protein(s). MEIG1/PACRG/motor complex transports cargos, including SPAG16L along the manchette to build sperm flagella. Other MEIG1-associated proteins, including TCFL5 and MORN3, might play a role in modulating the function of the MEIG1/PACRG complex.

Based on the findings described above and the reproductive phenotypes of the *Meig1* and *Pacrg* mutant mice, we propose the following model: MEIG1 and PACRG forms a complex in the manchette, this complex drives cargos, like SPAG16L, along the microtubules to the centrioles, where these cargos are used to assemble the sperm tail (Fig. 7). It is not clear how the MEIG1/PACRG complex is attached to the microtubules. It is unlikely that the complex directly binds to the microtubules, because both proteins are localized in the cytoplasm in transfected mammalian cells, not on the microtubule (W.L. and Zhibing Zhang, unpublished). Therefore, the binding must be mediated through other microtubule-binding protein(s), including motor proteins that provide energy for the movement. PACRG appears to be an upstream protein of MEIG1, even though MEIG1 stabilizes PACRG *in vitro*, it is unlikely MEIG1 is the protein that stabilizes PACRG *in vivo*. Thus, PACRG must be stabilized by other protein(s) *in vivo*, and these protein(s) might be involved in the connection of the MEIG1/PACRG complex to the microtubules. These proteins remain to be identified.

Besides PACRG and SPAG16L, MEIG1 also associates with transcription factor-like 5 protein (TCFL5) (Shi et al., 2013), and membrane occupation and recognition nexus repeat-containing 3 (MORN3) (Zhang et al., 2014). Interestingly, both TCFL5 and MORN3 are present in the manchette, and MORN3 localization is dependent on MEIG1 (Zhang et al., 2014). These proteins may modulate MEIG1/PACRG function. In addition, mouse *Spag16* gene encodes the full-length SPAG16L and a truncated SPAG16S protein; because the amino acids in SPAG16S are identical to the C terminus of SPAG16L, MEIG1 may also bind to SPAG16S. SPAG16S is believed to play a role in the regulation of spermatogenesis; it is not clear yet whether its function is related to the MEIG1/PACRG complex. It has been shown that intraflagella transport (IFT) is a key system for transporting cargo for ciliogenesis (Pedersen and Rosenbaum, 2008; Scholey and Anderson, 2006), and several IFT components are present in the manchette (Lehti et al., 2013; Kierszenbaum, 2002). Is there crosstalk between the MEIG1/PACRG complex and IFT? MEIG1 consists of only 88 amino acids and no known functional domains have been identified by bioinformatics analyses. How does this small protein associate with many different proteins? Besides SPAG16L, does the MEIG1/PACRG complex carry other cargos, and what are they? What motor proteins are involved in the transport? All these questions remain to be answered.

In summary, we have discovered a new transporting system in mouse elongating spermatids: the MEIG1/PACRG complex. This complex is responsible for driving cargos, such as SPAG16L, to build the sperm tail. Disrupting this transport system leads to failure of assembling sperm flagella and spermiogenesis arrest.

## MATERIALS AND METHODS

### *Meig1*, *Pacrg* and *Spag16L* mutant mice

*Meig1* and *Spag16L* mutant mice have been generated previously in our laboratory (Zhang et al., 2009, 2006), A spontaneous mutant mouse line carrying a *Pacrg* mutation was purchased from Jackson Laboratory (stock number: 000567). All animal work was approved by Virginia Commonwealth University's Institutional Animal Care and Use Committee (protocol AD10000167) in accordance with federal and local regulations regarding the use of non-primate vertebrates in scientific research.

### Histology

Sections of testis (7 μm) were prepared. H&E staining on mouse testes at indicated ages was carried out using standard procedures.

### Expression vector constructs

PACRG/pEGFPC1 was constructed previously (Zhang et al., 2009). To clone mouse *Pacrg* cDNA into pCS2+MT vector, full-length mouse *Pacrg* cDNA was amplified using the following primers: forward, 5′-GAATTCAAATGCCGAAGAGGACTAAACTG-3′; reverse, 5′-CTCGAGTCAGTTCAGCAAGCACGACTC-3′. After sequencing, the cDNA was cloned into the *Eco*R1/*Xho*1 site to create PACRG/pCS2+MT plasmid. The plasmid was transfected to mammalian cells to express Myc-tagged PACRG protein. To clone mouse *Pacrg* cDNA into pGBKT7 vector, full-length mouse *Pacrg* cDNA was amplified using the following primers: forward: 5′-GAATTCATGCCGAAGAGGACTAAACTG-3′; reverse, 5′-GGATCCGTCAGTTCAGCAAGCACGACTC-3′. The cDNA was cloned into the *Eco*R1/*Bam*H1 site to create PACRG/pGBKT7 plasmid.

### Yeast two-hybrid screen

A pre-transformed mouse Normalized Mate & Plate library (Clontech) was screened with the full-length mouse *Pacrg*-coding region as bait following the protocol provided in the kit.

### Generation of an anti-PACRG monoclonal antibody

Monoclonal PACRG antibodies were generated by Abmart (Shanghai, China), project number 12884-1, with the peptide sequence RRGIHDMLEHGG.

### Mixed germ cells isolation

Enzyme-dissociated testicular cells were prepared using a method described previously (Tsuneoka et al., 2006). Briefly, testes from an adult mouse were de-capsulated and placed in 5 ml DMEM containing 0.5 mg/ml collagenase IV (Sigma-Aldrich, St Louis, MO, USA) and 1.0 mg/ml DNase I (Sigma-Aldrich), and then incubated for 30 min at 32°C to dissociate testicular cells, then centrifuged for 5 min at 200 ***g***. Dispersed mixed testicular cells were fixed by 15 min incubation in 4% paraformaldehyde/PBS (containing 4% sucrose) at room temperature, then washed three times with PBS. Prior to plating, cells were re-suspended in 12 ml PBS and 50 µl of cell suspension was spread on SuperFrost/Plus microscope slides (Fisher Scientific, Pittsburgh, PA, USA) and allowed to air dry.

### DNA array analysis

GenomeStudioV2010.1 was used for processing the scanned images by applying background subtraction and quantile normalization to the bead-level data to obtain expression summaries and detection call *P*-values. Probes having a detection *P*-value less than 0.05 were considered present and absent otherwise. Because the detection call indicates whether expression can be reliably measured, probes called absent for all 12 arrays were removed, leaving 24,926 probes for gene expression analysis. To identify probes exhibiting differential expression between wild-type and *Meig1* mutant mice at day 22 and at day 28, for each day, probe-level two sample *t*-tests were performed. *P*-values from the *t*-tests were subsequently used in obtaining the false discovery rate using the q-value method. Array data have been deposited in NCBI's Gene Expression Omnibus (Edgar et al., 2002) with accession number GSE65247.

### RT-PCR

Total RNA was isolated from mouse testes with TRIzol (Invitrogen), the RNA was reversed transcribed and the cDNAs were used for PCR using a primer set specific for mouse *Pacrg*; forward, 5′-ATGCCGAAGAGGACTAAACTG-3′; reverse, 5′-CTCATAGGGAAACGTCATTTC-3′. As a control, PCR was also conducted to amplify mouse 18s RNA using primers: forward, 5′-TAACGAACGAGACTCTGGCAT-3′; reverse, 5′-CGGACATCTAAGGGCATCACAG-3′.

### Cell culture and transfection

For transient expression experiments, COS-1 cells were transfected with PACRG/pCS2+MT alone or together with MEIG1/pTarget, with or without proteasome inhibitor MG132. Forty-eight hours post-transfection, the cells were collected in RIPA buffer for western blot analysis. To generate PACRG stable cells, CHO cells were seeded at 1.5×10^6^ cells/100 mm dish and the next day transfected with 6 μg/dish of PACRG/pEGFP-C1 plasmid using X-tremeGENE Transfection Reagents (Roche). Twenty-four hours after transfection, the cells were supplied with fresh medium containing 1.5 mg/ml G418. Resistant clones were selected between 15 and 20 days and expanded. The G418 concentration was maintained at 1 mg/ml in the cell culture medium. The established PACRG stable cells were seeded into six-well plates and transfected with MEIG1/pTarget or empty pTarget plasmids. Forty-eight hours after transfection, cells were collected in RIPA buffer for western blot analysis.

### Immunofluorescence staining

Deparaffinized tissue slides and isolated mixed germ cells were permeabilized with 1% Triton X-100 for 5 min at 37°C, blocked for 1 h at room temperature with 10% goat serum in PBS. Following overnight incubation at 4°C with primary antibodies (1:200 dilution), slides were washed with PBS and incubated for 1 h at room temperature with Alexa 488-conjugated anti-mouse IgG secondary antibody (1:3000; Jackson ImmunoResearch Laboratories) or Cy3-conjugated anti-rabbit IgG secondary antibody (1:5000; Jackson ImmunoResearch Laboratories). Finally, slides were washed with PBS and sealed using VectaMount with 4′,6-diamidino-2-phenylindole (DAPI) (Vector Laboratories). Images were taken by confocal laser-scanning microscopy (Leica TCS-SP2 AOBS).

### Western blot analysis

Lysates collected from mammalian cells and testicular extracts were heated to 95°C for 10 min in sample buffer, loaded onto 10% sodium dodecyl sulfate-polyacrylamide gels, electrophoretically separated and transferred to polyvinylidene difluoride membranes (Millipore). Membranes were blocked [Tris-buffered saline solution containing 5% nonfat dry milk and 0.05% Tween 20 (TBST)] and then incubated with indicated antibodies at 4°C overnight. After washing in TBST, the blots were incubated with second antibodies for 1 h at room temperature. After washing, the proteins were detected with Super Signal chemiluminescent substrate (Pierce).

## Supplementary Material

Supplementary Material
