# Supplementary material for: A MEIG1/PACRG complex in the manchette is essential for building the sperm flagella
Source: Development. 2015 Mar 1;142(5):921–30. doi: 10.1242/dev.119834 (PMC4352978; doi:10.1242/dev.119834)
Supplement: Supplementary Material [file supp_142_5_921__index.html]

Supplementary Material 

# A MEIG1/PACRG complex in the manchette is essential for building the sperm flagella

## DEV119834 Supplementary Material

**Files in this Data Supplement:**

- Supplementary Material
