# Supplementary material for: A MEIG1/PACRG complex in the manchette is essential for building the sperm flagella
Source: Development. 2015 Mar 1;142(5):921–30. doi: 10.1242/dev.119834 (PMC4352978; doi:10.1242/dev.119834)
Supplement: Supplementary Material [file supp_142.5.921_DEV119834supp.pdf]

Table S1. Genes differently expressed in the testis between wild-type and *Meig1*-deficient mice at day 22 (A) and day 28 (B) after birth.

A: Day 22 after birth.

ProbeID	Symbol	Chromosome	wt	ko	Fold Change	P-value	FDR
ILMN_2493756	Try4		1.67	4.57	-7.45	0.000352545	1
ILMN_2721724	Dpp9	17	7.56	7.85	-1.22	0.000958527	1
ILMN_3058137	Mgat1	11	5.94	5.58	1.28	0.001524673	1
ILMN_2557918	1700020I14Rik		5.21	5.66	-1.36	0.002265999	1
ILMN_2964511	Pdcl3	1	4.32	5.03	-1.63	0.002975524	1
ILMN_1238805	A730011L01Rik	11	5.63	6.28	-1.56	0.003056812	1
ILMN_2732816	LOC674135	17	9.26	9.63	-1.29	0.005065892	1
ILMN_2651359	Zfp809	9	8.63	8.97	-1.27	0.005141298	1
ILMN_2545156	6330408J11Rik		6.98	6.16	1.77	0.005423706	1
ILMN_2729588	Sytl3	17	5.13	4.03	2.14	0.005901112	1
ILMN_2553710	A430010E21Rik		4.83	4.16	1.60	0.006073151	1
ILMN_3155626	EG245297	12	7.12	7.60	-1.39	0.006188792	1
ILMN_2723329	Cnksr1	4	0.89	4.37	-11.19	0.006382038	1
ILMN_3035378	Ankrd40	11	5.74	6.37	-1.55	0.006664827	1
ILMN_2481127	Zscan2	7	8.12	8.25	-1.09	0.007251246	1
ILMN_2588771	Fbxl8	8	5.39	5.99	-1.51	0.007401217	1
ILMN_2706231	Syngr2	11	8.45	8.68	-1.17	0.007414606	1
ILMN_3116570	Pdhb	14	8.38	9.47	-2.14	0.007740023	1
ILMN_2860242	Klhl24	16	6.07	5.72	1.27	0.008089334	1
ILMN_1257789	Serpinf1		6.02	5.52	1.41	0.00846656	1
ILMN_2938390	Ipmk	10	7.35	7.71	-1.28	0.008969136	1

ILMN_1233075	Ercc2		5.62	6.17	-1.46	0.00915521	1
ILMN_2938973	C330005M16Rik	7	8.11	7.82	1.22	0.009191306	1
ILMN_1216235	C030013F01Rik		5.70	4.38	2.50	0.009234731	1
ILMN_2594768	Tha1		6.25	6.59	-1.27	0.009499258	1
ILMN_2429178	6820402O18Rik		4.59	5.42	-1.78	0.009574978	1
ILMN_2699621	Dlk2	17	4.87	4.52	1.27	0.009584467	1

B: Day 28 after birth.

ProbeID	Symbol	Chromosome	wt	ko	Fold Change	P-value	FDR
ILMN_1239570	Elfn2	15	10.22	9.65	1.48	0.000466606	1
ILMN_2760671	D030056L22Rik		13.23	13.32	-1.06	0.000541726	1
ILMN_1231553	Bdh1	16	8.15	7.84	1.24	0.000576014	1
ILMN_1222970	F5	1	7.61	7.37	1.18	0.000716594	1
ILMN_1232951	EG434404	9	8.05	7.73	1.25	0.000882849	1
ILMN_1229266	Phf21b	15	7.30	7.52	-1.17	0.001199062	1
ILMN_1222474	Slc25a17		8.77	7.96	1.75	0.001359421	1
ILMN_2492488	4930527E20Rik		7.04	5.54	2.83	0.001372171	1
ILMN_1221102	Arl5a	2	5.32	0.78	23.20	0.001467126	1
ILMN_3104118	AW548124	17	8.43	8.95	-1.43	0.00148049	1
ILMN_2747037	Dkk1		5.36	3.71	3.15	0.001575625	1
ILMN_2771219	LOC669660		5.42	6.04	-1.54	0.001824839	1
ILMN_1256668	Fig4	10	2.12	3.61	-2.81	0.001857271	1
ILMN_2614351	Serhl	15	4.47	7.79	-10.00	0.00194424	1
ILMN_1237596	D030069G17Rik		6.00	5.38	1.53	0.001991942	1

ILMN_3150677	Sprr2a	3	6.55	5.99	1.47	0.001993995	1
ILMN_1219764	Msr2		2.24	5.17	-7.61	0.002097919	1
ILMN_2729374	Slc35c2	2	9.99	10.23	-1.19	0.002467506	1
ILMN_2901903	Pigf	17	12.66	12.50	1.12	0.002552619	1
ILMN_2695867	Hlcs		6.35	6.68	-1.25	0.002626226	1
ILMN_2608429	Atp6v0d1	8	10.01	10.26	-1.19	0.002733054	1
ILMN_2708303	Actn3	19	4.43	5.50	-2.10	0.003075408	1
ILMN_3009738	1600012H06Rik	17	6.97	6.66	1.25	0.003094901	1
ILMN_3160185	Defb23	2	5.67	0.36	39.78	0.003351663	1
ILMN_2773211	Kras	6	9.29	9.46	-1.12	0.003538508	1
ILMN_2849305	BC038613	17	5.53	5.96	-1.35	0.003558128	1
ILMN_2842654	Wdr31	4	11.41	11.24	1.13	0.003724702	1
ILMN_2646618	Fn1	1	0.00	1.84	-3.57	0.003753914	1
ILMN_2978585	Rbm18	2	7.01	7.16	-1.10	0.003837122	1
ILMN_2941324	Gsdmdc1	15	7.83	6.95	1.84	0.004134327	1
ILMN_2937674	Gse1	8	6.83	7.15	-1.25	0.004302601	1
ILMN_1221548	LOC385662		9.58	9.35	1.18	0.004490056	1
ILMN_1229960	Mcam	9	7.16	6.88	1.21	0.004490226	1
ILMN_1237895	Eif1ad	19	9.98	10.18	-1.15	0.004594248	1
ILMN_1223176	Magohb	6	6.53	6.20	1.25	0.004615987	1
ILMN_3090123	Dync2li1	17	10.52	10.42	1.08	0.004705032	1
ILMN_3053291	Xkr7	2	6.19	5.57	1.54	0.004713268	1
ILMN_1227526	9330177P18Rik		4.46	5.06	-1.51	0.004824099	1
ILMN_1214053	Zfp64	2	6.43	6.71	-1.22	0.004954124	1
ILMN_1216098	Zfp746	6	10.12	9.97	1.10	0.005160847	1

ILMN_1231923	Olfr694		7	4.86	5.30	-1.36	0.005258038	1
ILMN_2690677	Clca3			5.03	4.44	1.50	0.005333584	1
ILMN_2699531	Rgs10		7	10.48	10.10	1.30	0.005348749	1
ILMN_1244102	Abca14			10.05	9.88	1.12	0.00535151	1
ILMN_3147074	Pecam1		11	5.48	4.73	1.68	0.005395909	1
ILMN_2775514	Ext2			2.74	5.05	-4.96	0.005874434	1
ILMN_2549504	2810425O13Rik			6.56	6.16	1.32	0.005972131	1
ILMN_1225224	Ttc14			6.93	6.58	1.27	0.005976063	1
ILMN_2546905	LOC100044395			8.81	8.07	1.67	0.006265241	1
ILMN_1215398	B230326M20Rik			4.39	5.01	-1.53	0.006280205	1
ILMN_1239542	Raet1c		10	1.26	0.00	2.39	0.006341121	1
ILMN_1225386	2300009A05Rik		9	11.84	11.69	1.11	0.006407608	1
ILMN_2632839	1300007L22Rik			3.72	5.00	-2.42	0.006420978	1
ILMN_2692986	2810410P22Rik			4.93	0.00	30.57	0.006470469	1
ILMN_2974069	Slc35a3		3	5.47	6.01	-1.46	0.006501466	1
ILMN_1212636	Strap		6	13.08	13.26	-1.13	0.006668572	1
ILMN_2828677	Lgals8		13	9.04	8.72	1.25	0.006682785	1
ILMN_2722716	Atp1a2		1	7.66	7.32	1.26	0.006723269	1
ILMN_1242969	D030041G07Rik			4.63	5.07	-1.36	0.006763345	1
ILMN_1230145	Acvr2b		9	8.96	9.09	-1.09	0.006791532	1
ILMN_2613636	Col4a3bp		13	11.37	11.13	1.18	0.0071378	1
ILMN_1257637	LOC100045280			10.57	9.88	1.61	0.007279622	1
ILMN_2676606	Optn		2	11.13	11.57	-1.36	0.00735952	1
ILMN_1220039	Kif4	X		7.44	7.85	-1.33	0.007556084	1
ILMN_1237741	LOC383124			4.62	3.96	1.58	0.007735995	1

ILMN_1239224	Ccnt1			9.79	9.61	1.13	0.007927824	1
ILMN_1214648	C030024L24Rik			6.77	6.43	1.27	0.008067697	1
ILMN_1220376	1700007N14Rik			10.35	9.79	1.47	0.00812489	1
ILMN_2997406	Arl4c		1	7.09	6.57	1.43	0.008137997	1
ILMN_2517100	Pgm2l1			6.84	6.57	1.21	0.008152468	1
ILMN_1237842	Clk2		3	7.19	7.65	-1.37	0.00828342	1
ILMN_1242799		6-Mar	15	10.83	10.52	1.24	0.008443754	1
ILMN_1252601	Bcl7a		5	6.20	5.92	1.21	0.008507931	1
ILMN_1218116	Bmp1		14	6.50	6.89	-1.31	0.008557226	1
ILMN_2449449	Zfp68		5	6.25	5.61	1.56	0.008660074	1
ILMN_2715664	Atf1			5.45	4.81	1.56	0.008688119	1
ILMN_1223847	Chk			5.49	4.95	1.46	0.00902846	1
ILMN_1237670	Entpd2		2	8.54	8.21	1.26	0.009059968	1
ILMN_1226636	scl00008.1_398			5.98	5.46	1.43	0.009127992	1
ILMN_2832105	Fgg		3	6.36	5.82	1.46	0.009341256	1
ILMN_2897328	Zfp777		6	5.19	4.55	1.55	0.009354505	1
ILMN_1245733	Ddx49		8	5.58	6.02	-1.36	0.00947922	1
ILMN_2578237	Mbc2			5.01	5.94	-1.91	0.009858187	1
ILMN_1253145	Tpd52		3	5.16	4.49	1.58	0.009923179	1

Notice that not common genes were found at the two time points, verification for some of these genes (i.e., *Arl5a*) by q-PCR and Western blot analysis showed no differences between wild-type and *Meig1*-deficient mice. The few differences in expression levels between wild-type and *Meig1*-deficient mice detected by DNA microarray studies may reflect an artifact arising from low baseline levels of expression. *Meig1* gene was not identified as no *Meig1* probes were present in the arrays.

Supplemental Figures

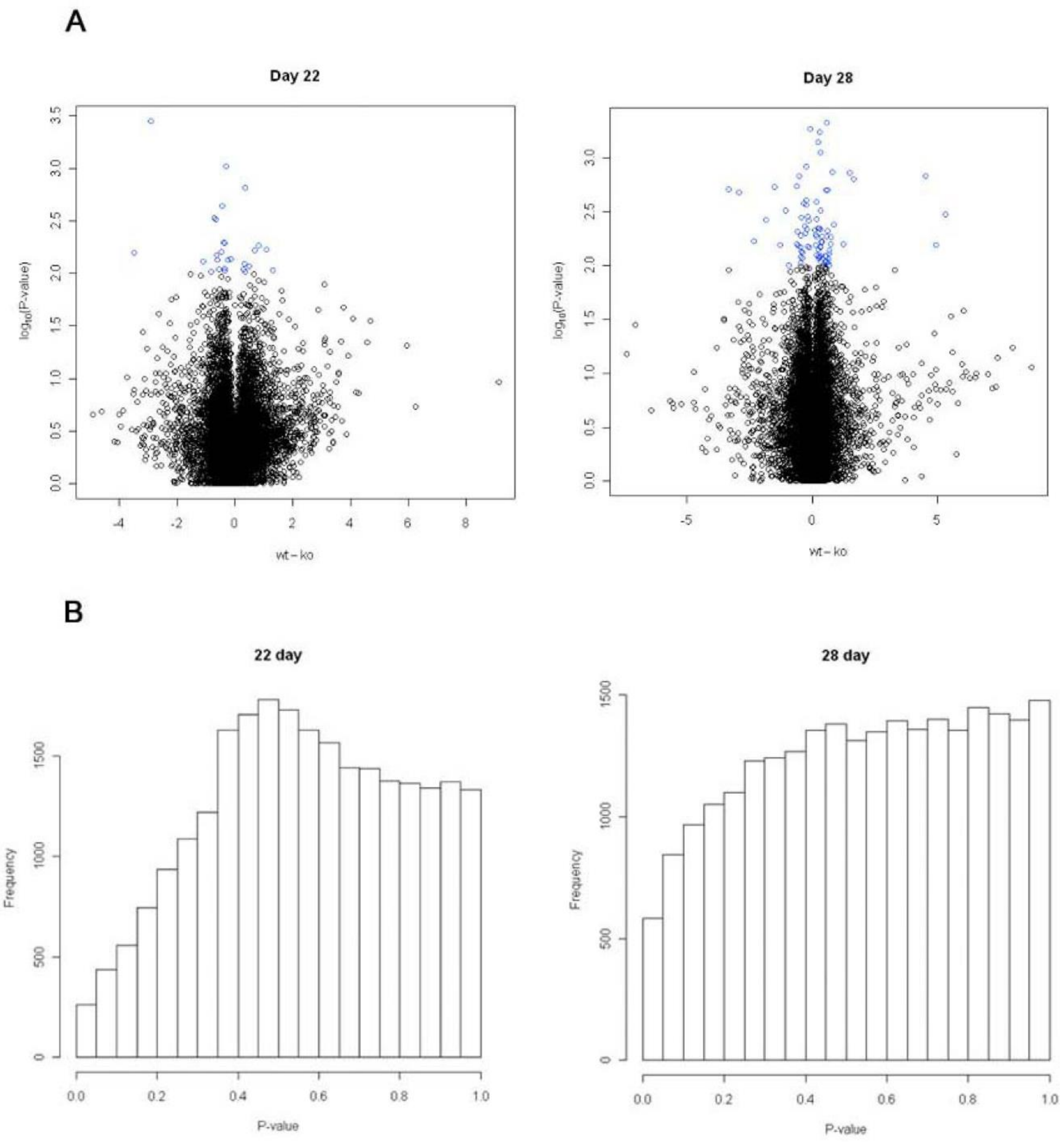

Supplemental Figure 1. Comparison of gene expression profiles between wild-type and *Meig1*-deficient mice by DNA microarray.

A. Volcano plot from the wild-type vs *Meig1*-deficien mice comparison where the difference between average expression between wild-type and mutant mice is plotted on the x-axis and $-\log_{10}$ (P-value) is plotted on the y-axis. Blue points correspond to probe sets significant using a P<0.01 threshold. Top panel: Day 22; bottom panel: Day 28.

B. Histogram of p-values from two-sample t-test comparing wild-type to mutant mice which demonstrates the large number of significant probes contribute to very high observed FDRs (minimum FDR was 1). Top panel: Day 22; bottom panel: Day 28.

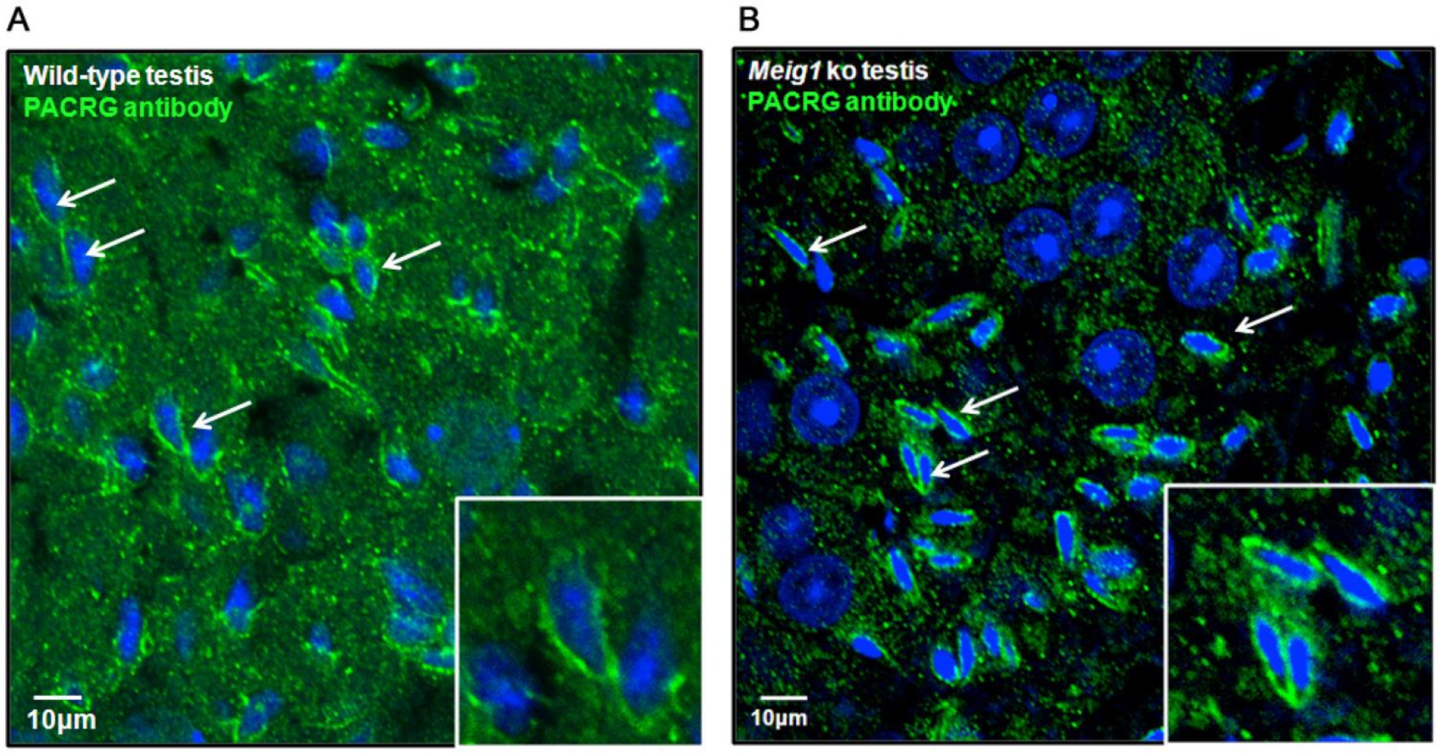

Supplemental Figure 2. PACRG localization in wild-type and *Meig1*-deficient mouse testis.

A. Testicular sections from adult wild-type mice were stained with a specific PACRG antibody, specific signal was detected in the post-meiotic germ cells, and the signal was around nuclei of elongating spermatids. The insert shows zoomed-in spermatids.

B. Testicular sections from adult *Meig1*-deficient mice were stained with an anti-PACRG antibody, notice that PACRG is still localized in the region around nuclei (arrows).

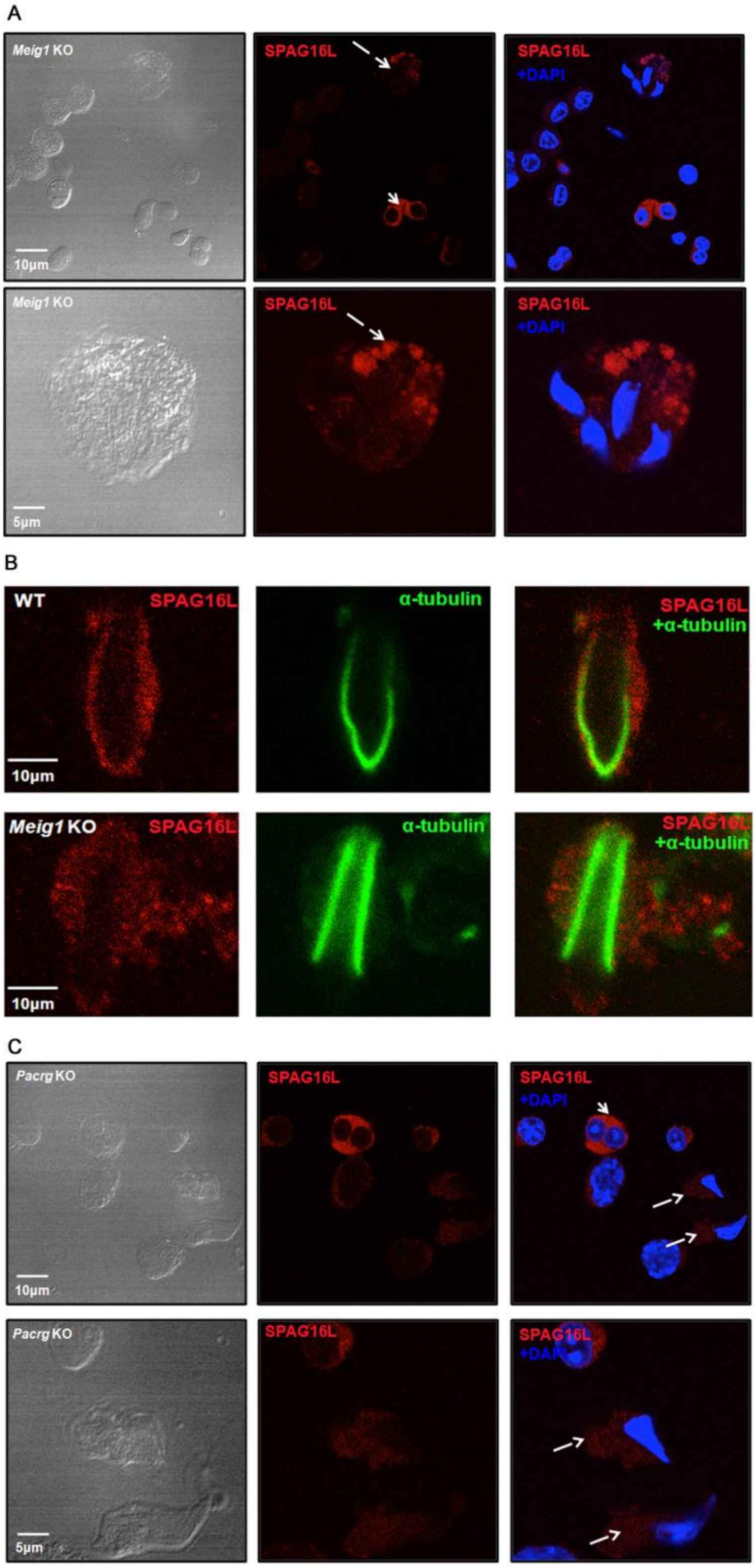

Supplemental Figure 3. SPAG16L fails to localize in the manchette in the elongating spermatids of *Meig1* or *Pacrg*-deficient mice.

A. Low magnification images (upper panel) of mixed germ cells from *Meig1*-deficient mice stained with an anti-SPAG16L antibody. The arrowhead points to round spermatids, where SPAG16L is still present in the

cytoplasm. The dashed arrow points to clusters of remaining elongating spermatids. Notice that SPAG16L is still present in the whole cytoplasm. The lower panel shows the zoomed-in images of the remaining elongating spermatids. B. High magnification images showing an elongating spermatid from a wild-type mouse (upper panel) and a *Meig1*-deficient mouse (lower panel). Notice that SPAG16L is present in the manchette of the elongating spermatid from the wild-type mouse, but its localization is diffused in the remaining elongating spermatid from the *Meig1*-deficient mouse. C. SPAG16L localization in the elongating spermatids of *Pacrg*-deficient mice. Notice that SPAG16L is present in the whole cytoplasm of the remaining elongating spermatids (arrows). The arrowhead points to round spermatids, where SPAG16L is still present in the cytoplasm, the same localization as in the wild-type mice.

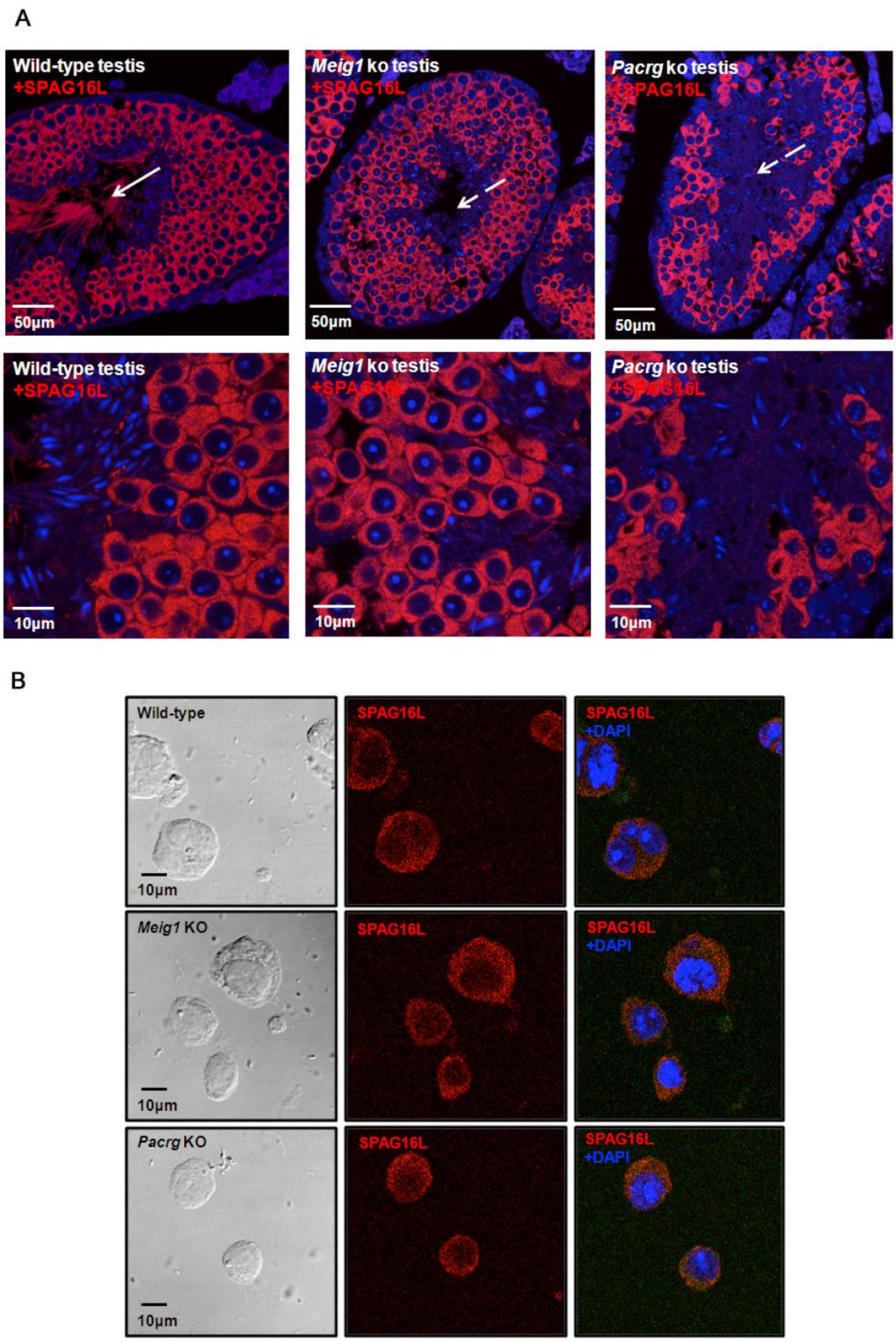

Supplemental Figure 4. SPAG16L localization is not altered in early germ cells of *Meig1* and *Pacrg*-deficient mice.

A. Testicular sections, B. Mixed germ cells. Notice that SPAG16L was localized in the cytoplasm of spermatocytes/round spermatids from all the three types of mice. The arrow in 4A points to released sperm in the lumen of seminiferous tubule of a wild-type mouse, the dashed arrows point to the empty lumen of seminiferous tubules of a *Meig*1 and a *Pacrg*-deficient mouse.
